# Adaptive Responses of Large Yellow Croaker *Larimichthys crocea* to Ocean Acidification: Integrative Analysis of Gill and Kidney Transcriptomics and Antioxidant Enzyme Activities

**DOI:** 10.3390/antiox14070872

**Published:** 2025-07-16

**Authors:** Ting Ye, Xiaoyan Zhang, Feng Liu, Xiao Liang, Dandan Guo, Bao Lou, Zhigang Xie

**Affiliations:** 1 State Key Laboratory for Managing Biotic and Chemical Threats to the Quality and Safety of Agro-Products, Institute of Hydrobiology, Zhejiang Academy of Agricultural Sciences, Hangzhou 310021, China; 15tye@stu.edu.cn (T.Y.); liufeng@zaas.ac.cn (F.L.); liangx@zaas.ac.cn (X.L.); guodd@zaas.ac.cn (D.G.); 2 Zhejiang Key Laboratory of Coastal Biological Germplasm Resources Conservation and Utilization, Wenzhou 325005, China; 3 College of Biological and Environmental Sciences, Zhejiang Wanli University, Ningbo 315000, China; 2023881053@zwu.edu.cn

**Keywords:** acidification, adaptive, *Larimichthys crocea*, gill, kidney, antioxidant, transcriptomic

## Abstract

Anthropogenic acidification is a long-term challenge to marine ecosystems. Though coastal acidification is intensifying, the large yellow croaker (*Larimichthys crocea*) exhibits good adaptability to pH fluctuations, the underlying mechanisms of which remain poorly understood. This study investigated the morphology, antioxidant enzyme activity, and gene expression of *L. crocea* under varying acidification conditions (pH 8.1 (H group), 7.8 (M group), and 7.4 (L group)). Water pH fluctuations were also monitored to explore the physiological responses and potential adaptive molecular mechanisms of *L. crocea* under various acidified environments. The results indicated that the water pH decreased in the H group, significantly increased in the L group (*p* < 0.05), and remained stable in the M group during the experiment. The lowest MDA content and the highest antioxidant enzyme activities (CAT, SOD, GSH-Px) were observed in *L. crocea* at pH 7.8, suggesting pH 7.8 was optimal for *L. crocea*. Transcriptomic analysis revealed distinct gene expression patterns between the gills and kidneys under acidification stress. Differentially expressed genes (DEGs) in the gills were primarily observed between the M and L groups (62.3%), whereas in the kidneys, the majority of DEGs were observed between the M and H groups (43.2%). These findings suggested that the gills play a critical role in adapting to low pH in *L. crocea*, while the kidneys were more responsive to high pH. Enrichment analysis identified critical pathways, including vasopressin-regulated water reabsorption, mineral reabsorption, and aldosterone-regulated sodium reabsorption, which are associated with water and ion metabolism. These pathways play a pivotal role in the acid–base homeostasis and metabolism of *L. crocea*. These results provide insights into the adaptive mechanisms of *L. crocea* to acidified environments, with implications for aquaculture management and future ocean acidification adaptation.

## 1. Introduction

Anthropogenic activities, such as the combustion of fossil fuels, produce large amounts of CO_2_, approximately one-third of which is absorbed by the ocean [1]. This leads to rapid changes in the seawater carbonate system and pH, a process known as “ocean acidification” [2]. As global climate change intensifies and human activities continue to increase, ocean acidification has become an increasingly severe issue [3]. The suitability of aquatic environments is crucial for the survival, growth, and reproduction of fish [4], and exposure to acidified water can increase the allostatic load on marine fish [5]. Studies have shown that ocean acidification interferes with fish sensory functions, including olfaction [6], hearing [7], and vision [8], and may lead to oxidative stress responses and disruptions in energy metabolism [9,10,11,12]. However, some studies suggest that fish, as aquatic vertebrates, possess a strong ability to regulate ionic balance, which enables them to partially withstand the negative impacts of ocean acidification [13,14,15,16]. Though the specific effects of ocean acidification on fish remain controversial [17,18,19], there is a general consensus that the impact of ocean acidification on marine ecosystems varies significantly among species [20].

In addition to the impacts on global ocean acidification, coastal waters are also subject to the combined influences of various factors, such as the discharge of domestic and agricultural wastewater, eutrophication, acid rain, and groundwater runoff [21,22,23]. These factors further exacerbate the extent of seawater acidification. Currently, the acidification level in coastal waters will reach the global ocean acidification level by 2100 [24,25,26]. Fish living in these coastal environments are exposed to more drastic pH fluctuations, suggesting that they have evolved more efficient adaptive traits to cope with the challenges of low pH conditions [15]. Investigating the molecular mechanisms underlying these adaptive traits will provide valuable insights into how fish might adapt to prolonged ocean acidification in the future. The large yellow croaker (*Larimichthys crocea*), a member of the family Sciaenidae in the order *Perciformes*, is renowned for its golden color and delicate flesh, which is ranked first among the “Four Major Sea Products” of the East China Sea. It is widely distributed in the East Sea and South Sea of China, the southern coastal areas of the Korean Peninsula and Japan [27]. Since the successful development of artificial breeding in 1985, the large yellow croaker has achieved remarkable success in offshore fishing in China, and has rapidly become the most widely produced marine fish species, with a production of 0.28 million tons in 2023 [28]. Surprisingly, according to our preliminary annual survey of aquaculture areas for *L. crocea*, within the densely populated aquaculture zones, the surface water (1~1.5 m) pH fluctuated rapidly between 7.5 and 8.0 throughout the year (data not yet published), indicating a high degree of acidification. In initial acidification stress experiments, we observed that when *L. crocea* were exposed to low pH water, the pH of the water significantly increased within 12 h. These observations suggested that *L. crocea* likely engages in a substantial material exchange with its external environment to maintain an appropriate pH for its survival, indicating their good adaptation to acidic environments. However, our knowledge of the molecular mechanisms underlying this adaptive response remains limited.

To cope with the transitions between diverse habitats, vertebrates have evolved efficient acid excretion mechanisms to maintain the acid–base balance in their body fluids [14]. Fish are primarily ammoniotelic, and as such they have developed a flexible strategy to counteract osmotic pressures resulting from environmental acidification, alkalinization, and ion concentrations changes, via regulation of acid–base equivalents (H^+^, NH_4_^+^, OH^3−^, and HCO_3_^3−^), as well as ammonia and phosphate [29,30,31]. Gills in fish are primary organs for gas exchange, ion regulation, acid–base balance, and nitrogenous waste excretion, by which over 90% of the acid–base regulation in body fluids is handled [16,32,33]. Although kidneys in fish play a less prominent role in overall acid–base excretion than gills, they still serve an indispensable auxiliary function in acid–base regulation [31]. Notably, the kidneys express carbonic anhydrases and HCO_3_^3−^ transporters, which further regulate the acid–base balance of the entire body by controlling the reabsorption of HCO_3_^3−^ in the filtrate [34,35,36]. Previously, studies have extensively documented that fish maintain internal pH stability under acidification stress through branchial and renal ion exchange, respiratory regulation, and blood buffering [13,14,15,16]. Given the central roles of the gills and kidneys in acid–base regulation, this study focuses on these two organs to explore the mechanism of acidification adaptation in fish by setting different pH of culture environments.

In order to adapt to environments with decreasing pH, marine organisms must adjust their physiological functions, which is achieved at the molecular level through gene expression [37]. With the rapid development of next-generation sequencing technologies, transcriptome sequencing has become a key tool for studying gene expression [38]. By utilizing RNA sequencing, we can reveal the molecular network responses related to the adaptation of *L. crocea* under different acidic conditions at the transcriptional level. Using *L. crocea* as a model organism, this study simulates projected ocean acidification scenarios in 2100 (pH 7.7–7.8) and 2300 (pH 7.3–7.4) to conduct acidification stress experiments [39]. By monitoring changes in culture water pH, and analyzing tissue structure and redox enzyme responses, this study first investigated the adaptability of *L. crocea* under different acidification conditions. Furthermore, transcriptomic analyses of the gills and kidneys were performed to uncover the molecular mechanisms underlying the acidification adaptation in *L. crocea*. Our findings will not only contribute to the knowledge of acidification adaptation mechanisms of organisms, but also provide important insights for optimizing aquaculture management strategies under ocean acidification conditions.

## 2. Materials and Methods

### 2.1. Chemicals

HCl (purity 36%) and NaHCO_3_ were purchased from Sinopharm Reagent Co., Ltd. (Beijing, China).

### 2.2. Animals

The *L. crocea* used in this study were sourced from the aquaculture population of Zhejiang Xiangshan Harbor Aquatic Seedling Co., Ltd. (Ningbo, Zhejiang, China). The animal experimental procedures were approved by the Ethics Committee of the Institute of Animal Science, Zhejiang Academy of Agricultural Sciences, China (Approval No. 2023ZAASLA33). A total of 180 fish, aged 7 months (average length: 11.6 ± 0.5 cm, average body weight: 13.92 ± 0.73 g), were acclimatized in a 2-ton tank for two weeks to adapt to the laboratory environment. During this period, the water temperature was maintained at 20 ± 1 °C, salinity at 24 ± 0.5, pH at 8.02 ± 0.06, with continuous aeration, daily water changes, and a light cycle of 12 h light and 12 h dark. The fish were fed commercial feed twice daily, and no mortality was observed during the acclimation period.

### 2.3. Experimental Design

A total of 180 fish were randomly assigned to three experimental groups. The medium acidification group (M) was exposed to pH 7.8, simulating ocean acidification conditions projected for 2100, while the low acidification group (L) was exposed to pH 7.4, simulating conditions for 2300 [39]. The control group (H) was maintained at pH 8.1, representing the current level of ocean acidification [2]. All groups were maintained under identical rearing conditions, with the only variable being the water pH. Each group of 60 fish was equally distributed into 3 300 L tanks, with 20 fish per tank and approximately 270 L of water per tank. A total of 9 tanks were utilized, arranged randomly to minimize potential confounding environmental factors, such as light and sound. Prior to the experiment, seawater was adjusted with equimolar amounts of HCl and NaHCO_3_ to ensure the desired pH conditions and stability [7,40]. The pH of the experimental water was measured twice daily using a YSI (ProQuatro, Yellow Springs, OH, USA) to confirm stability before transferring the fish into the tanks. Throughout the experiment, the water was replaced every two days to maintain water quality, and pH was adjusted before each water change using the bucket overturning method. Significant changes in water pH were observed 12 h after each water change, indicating intense physiological activities related to acid–base regulation in *L. crocea*. After three consecutive water changes, a consistent pH changes pattern was established. Samples were then collected 12 h after the third water change (132 h into the experiment). The total duration of the experiment was 6 days, with no fish mortality observed throughout.

### 2.4. Sample Collection

Six fish were randomly selected from each tank, resulting in a total of eighteen fish samples per group. The remaining experimental fish were returned to the holding tanks and carefully maintained. After anesthetization with MS-222 (methanesulfonate-222), the fish were placed on ice [41]. Tissue samples, approximately 1 cm in size, were excised from the central region between the second gill arch and the kidney using sterile surgical scissors, and fixed in 10 volumes of 4% paraformaldehyde [42]. The gill filaments and kidneys were then carefully rinsed with pre-chilled saline. Tissue from every 3 fish was pooled to form one biological replicate. All samples were immediately snap-frozen in liquid nitrogen and stored at −80 °C for subsequent enzyme activity assays, transcriptome sequencing, and qRT-PCR analysis.

### 2.5. Histological Observations

Histological examination was performed according to the method described in our previous study [42]. Briefly, gills and kidneys were dehydrated through a series of alcohol gradients and then embedded in paraffin. The samples were sectioned into 7 μm thick slices along the direction of the gill filaments and the largest cross-sectional area of the kidney using a Shannon rotary microtome (Thermo Fisher Scientific, Waltham, MA, USA). The sections were then stained with hematoxylin and eosin (H&E). Finally, the stained sections were mounted on glass slides and examined under a light microscope at 200× magnification.

### 2.6. Oxidative Stress Enzyme Activities

The detection of antioxidant biomarkers was performed using commercially available kits and a UV-visible spectrophotometer (Unico, Dayton, NJ, USA). These biomarkers included superoxide dismutase (SOD, No. A001-3-2), glutathione peroxidase (GSH-Px, No. A005-1-2), catalase (CAT, A007-2-1), and malondialdehyde (MDA, No. A003-1-2), all purchased from Nanjing Jiancheng Bioengineering Institute (Nanjing, China).

The gill and kidney tissues were homogenized in pre-chilled phosphate-buffered saline. The homogenate was then centrifuged at 6000 rpm for 20 min at 4 °C, and the supernatant was collected for the determination of antioxidant activity and peroxide product levels. CAT activity was measured according to Aebi [43] by monitoring the decrease in absorbance at 240 nm due to H_2_O_2_ consumption. One unit of CAT is defined as the amount of enzyme required to reduce 1 μmol of H_2_O_2_ min^−1^. SOD activity was measured using the nitroblue tetrazolium reduction inhibition assay, with xanthine and xanthine oxidase as the superoxide generating system, and a detection wavelength of 450 nm [44]. GSH-Px concentration was determined using the method of Tietze [45] at a wavelength of 405 nm. MDA content was quantified using the method described by Ohkawa et al. [46], which involves the reaction of MDA with thiobarbituric acid to form a red product, with maximum absorbance measured at 532 nm. All measurements were normalized to the protein content in the supernatant, with protein concentration determined by the Lowry method [47].

### 2.7. RNA Extraction and Sequencing

Total RNA from the gill and kidney tissues was extracted using the Total RNA Purification Kit (Omega, Mountain Lakes, NJ, USA), and quality control was performed using 0.8% agarose gel electrophoresis and NanoDrop 2000 (Thermo Fisher Scientific, Waltham, MA, USA). For each pH treatment group, 9 gill/kidney samples were pooled, with 3 samples of equal molar mass per pool, resulting in 3 biological replicates for RNA-Seq. Sequencing was conducted on the Illumina MiSeq platform (Personal Biotech Co., Ltd., Shanghai, China). Raw data were filtered using the FastQC tool, and clean reads were aligned to the reference genome of the large yellow croaker, Daiqu strain (PRJNA1168280), using HISAT2. The mapped genes were annotated against the Pfam, SUPERFAMILY, GO, and KEGG databases. Gene abundance was estimated for each sample using RSEM and normalized to reads per kilobase per million reads (FPKM) to obtain gene expression levels. Additionally, differentially expressed genes (DEGs) were identified using DESeq2 1.48.1 software. The thresholds for significant DEGs were set as |log_2_(fold change)| ≥ 1 and *p* < 0.05.

### 2.8. Validation of Sequencing Data

To validate the accuracy of the sequencing results, we selected 10 DEGs for qRT-PCR analysis. These genes were involved in the phagosome, adipocyte lipolysis regulation, and TNF signaling pathway. β-actin was used as the reference gene. Gene-specific primers (Supplementary Table S1) were designed using Primer 5.0 software (Premier Biosoft, San Francisco, CA, USA). The qRT-PCR reactions were performed in a total volume of 20 μL, consisting of 1 μL cDNA, 10 μL 2 × SYBR real-time PCR mix (Tiangen Biochemical Technology Co., Ltd., Beijing, China), 1 μL primers, and 7 μL ddH_2_O. The PCR program included an initial denaturation at 95 °C for 5 min, followed by 40 cycles of denaturation at 95 °C for 15 s, annealing at 60 °C for 30 s, and extension at 72 °C for 15 s. Three technical replicates were performed for each sample. Fluorescent signals were recorded using the QIAquant 96 2plex (QIAGEN, Hilden, Germany). The relative expression levels of the target genes were calculated using the 2^3−△△Ct^ method, with β-actin as the reference gene. By comparing the fold changes obtained from RNA-Seq and qRT-PCR analyses, we confirmed the gene expression patterns of key and hub genes. The consistency of the gene expression trends further validated the accuracy of the RNA-Seq results.

### 2.9. Statistical Analysis

In this study, data are presented as the mean ± standard deviation (SD) (mean ± SD) for each group. Normality and homogeneity of variance were assessed using SPSS 19.0 (IBM, Armonk, NY, USA). One-way analysis of variance (ANOVA) was then performed to evaluate the effects of water pH, enzyme activity, and gene expression levels across the different groups. Statistical significance was set at a *p*-value < 0.05, with significant differences denoted by an asterisk (*) in the figures. For clarity and visual representation, experimental data were graphically displayed using GraphPad Prism 5.0 (GraphPad Software, San Diego, CA, USA).

## 3. Results

### 3.1. pH Fluctuations in Water Under Acidic Stress

**Figure 1:**
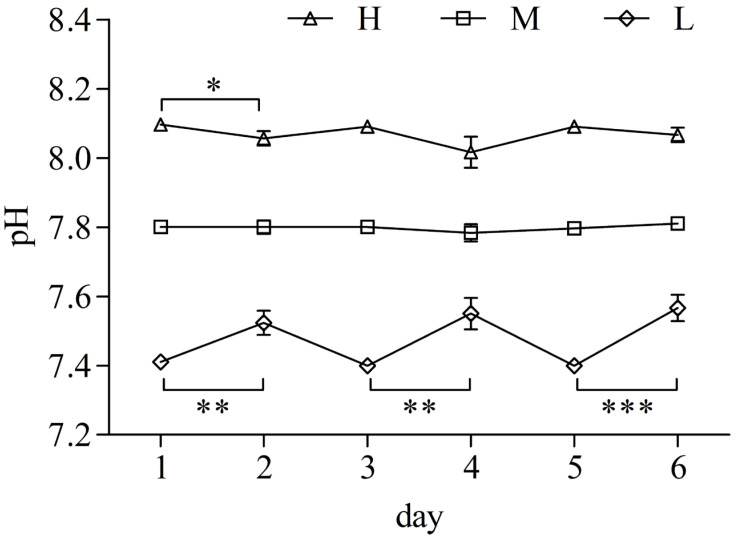
Changes in water pH across experimental groups under different acidification stress cycles. Days 1, 3, and 5 represent the pH conditions artificially adjusted after water changes. Results are expressed as mean ± SD. “*”, “**”, and “***”, indicating statistically significant differences between different groups. “*”, “**”, and “***” represent *p* < 0.05, *p* < 0.01, and *p* < 0.001, respectively.

To ensure water quality, the water was changed every other day throughout the experiment. However, we observed distinct patterns of pH fluctuation in the water after each water change across the three groups (Figure 1). In the pH 8.1 group (H), the pH decreased following water changes, with a significant reduction observed after the first change (*p* < 0.05). The pH of the pH 7.8 group (M) remained relatively stable throughout the experiment. In contrast, the pH of the pH 7.4 group (L) increased significantly after each water change (*p* < 0.01). These results suggest that *L. crocea* in the L and H groups may have engaged in acid–base ion exchange with the surrounding water, with the fish in the L group displaying a particularly strong physiological response in terms of acid–base regulation.

### 3.2. Histology Examination

**Figure 2:**
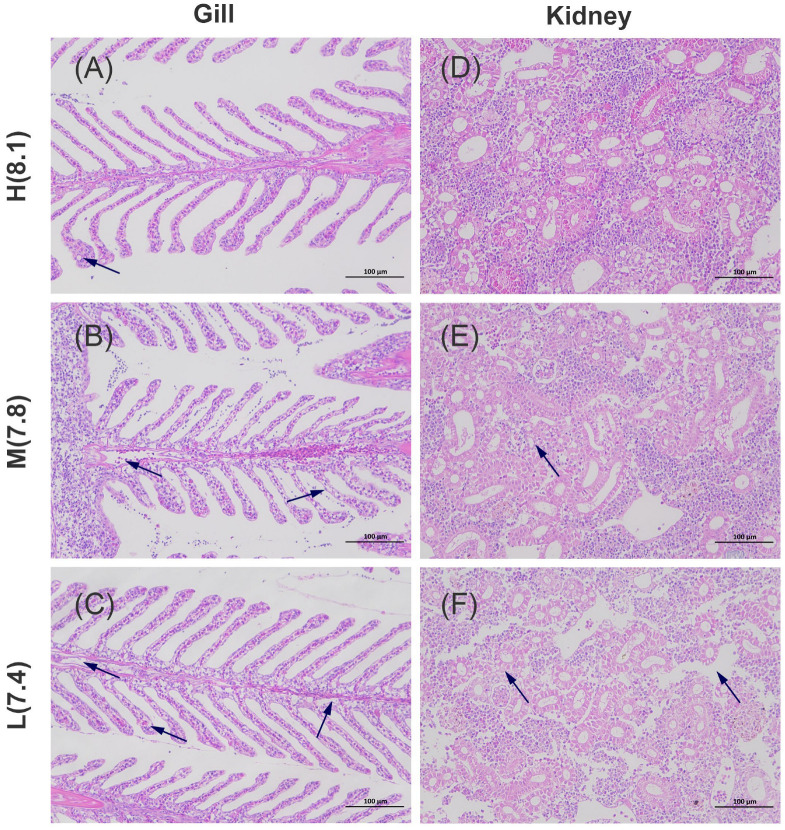
Histological sections of the gill and kidney tissues of *L. crocea* under acid stress (200× magnification, HE staining). (**A**) Gill section of the H group; (**B**) Gill section of the M group; (**C**) Gill section of the L group; (**D**) Kidney section of the H group; (**E**) Kidney section of the M group; (**F**) Kidney section of the L group. Black arrows indicate the locations of distinct structural abnormalities in the gill and kidney tissues.

The gill structure of *L. crocea* is similar to that of other teleost fish, consisting of gill filaments, secondary lamellae, and water channels. The gill filaments are covered by a dense layer of epithelial cells and contain chloride cells, lymphocytes, macrophages, and eosinophilic granulocytes. The secondary lamellae are primarily composed of pillar cells, squamous epithelial cells, and mucus-secreting cells. In the H group, the gill filament structure remains relatively intact, with only minimal vacuolation observed in the pillar cells of secondary lamellae (Figure 2A, black arrow). In the M group, epithelial cell shedding was observed in the blood vessels of the gill filaments, accompanied by pronounced vacuolation in the secondary lamellae (Figure 2B, black arrow). In the L group, similar epithelial cell shedding was noted in the blood vessels of the gill filament, with a significant number of chloride cells undergoing apoptosis (Figure 2C, black arrow). Regarding the kidneys, in the H group, the normal glomeruli and Bowman’s space are clearly visible, with intact structures of both the proximal and distal renal tubules (Figure 2D). In the M group, the glomerular structure appears loose, and the renal tubules exhibit cloudy swelling and degeneration (Figure 2E, black arrow). In the L group, Bowman’s space is absent, the renal tissue structure is markedly loose, and the renal tubules show signs of hyaline degeneration (Figure 2F, black arrow).

### 3.3. Variation of Antioxidant Enzyme Activities

**Figure 3:**
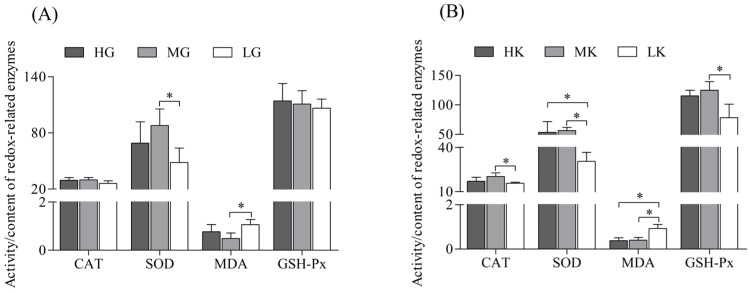
The activity of catalase (CAT), superoxide dismutase (SOD), glutathione peroxidase (GSH-Px), and malondialdehyde (MDA) content in the gills (**A**) and kidneys (**B**) of *L. crocea* under different pH conditions. Groups are as follows: gills (HG: pH 8.1, control; MG: pH 7.8; LG: pH 7.4) and kidneys (HK: pH 8.1, control; MK: pH 7.8; LK: pH 7.4). The *y*-axis represents enzymes’ activity or content, with the following units: CAT in U/mg protein^−1^, SOD in U/mg protein^−1^, GSH-Px in units, and MDA in nmol/mg protein^−1^. Results are expressed as mean ± SD (*n* = 6). “*” above the lines indicates significant differences between groups (*p* < 0.05).

Figure 3 illustrates the changes in redox enzyme systems in the gills and kidneys of *L. crocea* under different pH conditions. In the gills (Figure 3A), the activities of CAT and GSH-Px showed no significant differences across the three pH treatments, with changes considered negligible. SOD activity followed the order of MG > HG > LG, while MDA content exhibited the reverse pattern, MG < HG < LG, with significant differences observed only between the MG and LG groups (*p* < 0.05). In the kidneys (Figure 3B), the activities of CAT and GSH-Px were highest in the MK group, followed by HK and LK, with MK being significantly higher than LK (*p* < 0.05). Additionally, the LK group exhibited significantly lower SOD activity and significantly higher MDA content compared to both the HK and MK groups (*p* < 0.05). No significant difference was observed between the HK and MK groups. These findings suggest that under pH 7.4 conditions (L group), the redox enzyme systems in both the gills and kidneys of *L. crocea* are in a distinctly unbalanced state, whereas under pH 7.8 conditions (M group), the redox enzyme systems in both tissues are in a healthier state, even outperforming those under pH 8.1 conditions (H group, Control).

### 3.4. Sequencing Results and Differential Expression Analysis

High-throughput sequencing technology was employed to sequence the transcriptomes of the gill and kidney tissues of *L. crocea*. As shown in Table 1, the clean data from all samples exceeded 5.4 Gb, with Q30 values greater than 95.6% and GC content ranging from 43.76% to 48.01%, indicating that the sequencing results are highly reliable.

**Figure 4:**
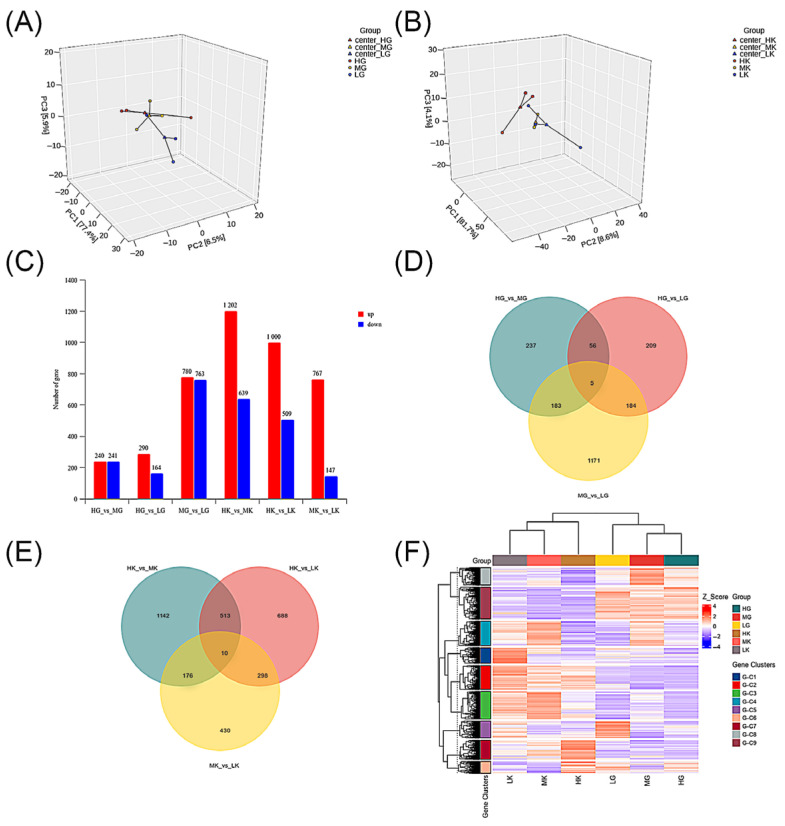
Analysis of gene expression in gill and kidney tissues under different pH conditions. Groups: gills (HG: pH 8.1, control; MG: pH 7.8; LG: pH 7.4) and kidneys (HK: pH 8.1, control; MK: pH 7.8; LK: pH 7.4). (**A**) Principal component analysis (PCA) of gene expression levels in gill. (**B**) PCA of gene expression levels in kidney. (**C**) Venn diagram for DEGs in gill. (**D**) Venn diagram for DEGs in kidney. (**E**) Statistical analysis of DEGs results; red indicates upregulation and blue indicates downregulation. (**F**) Cluster heatmap of DEGs, blue to red indicates high to low relative expression levels.

Principal component analysis (PCA) based on gene expression revealed that the LG group clustered separately, while the HG and MG groups did not fully separate (Figure 4A). In the kidneys, the HK group clustered independently, whereas the MK and LK groups did not completely separate (Figure 4B). This may suggest that the gene expression network in the kidneys is more sensitive to acidification, while the gills only respond under higher levels of acidification. Differentially expressed genes (DEGs) between different groups of gill and kidney tissues were identified using the thresholds of |log_2_(fold change)| ≥ 1 and *p* < 0.05. As shown in Figure 4C, in the gills there were 481 DEGs between the HG and MG groups, 454 DEGs between the HG and LG groups, and 1543 DEGs between the MG and LG groups (62.3% of DEGs in gills), with the latter being significantly higher than the others. In the kidneys, there were 1841 DEGs between the HK and MK groups (43.2% of DEGs in kidneys), 1509 DEGs between the HK and LK groups, and only 914 DEGs between the MK and LK groups, which was significantly lower than in the other groups; however, the differences in upregulated and downregulated genes were the most pronounced. Compared to the gills, gene expression in the kidneys is more influenced by pH, with a significant upregulation of genes in the higher pH groups. Moreover, the Venn diagram results indicated that, in the comparison across three different pH gradients, only five genes were co-expressed between all three groups in the gills (Figure 4D), with the majority of genes independently expressed between the MG and LG groups (1171 genes). The number of co-expressed genes between the HG and MG groups, as well as between the HG and LG groups, was the smallest (61 genes). In the kidneys (Figure 4E), only 10 genes were co-expressed between the three groups, with the HK and MK groups, as well as the MK and LK groups, showing the fewest co-expressed genes (186 genes). The clustering heatmap demonstrated that the expression patterns of DEGs were entirely different in the gills and kidneys, suggesting that the responses to acidification in the two tissues may involve distinct mechanisms (Figure 4F).

### 3.5. KEGG Pathway Enrichment Based on DEGs

**Figure 5:**
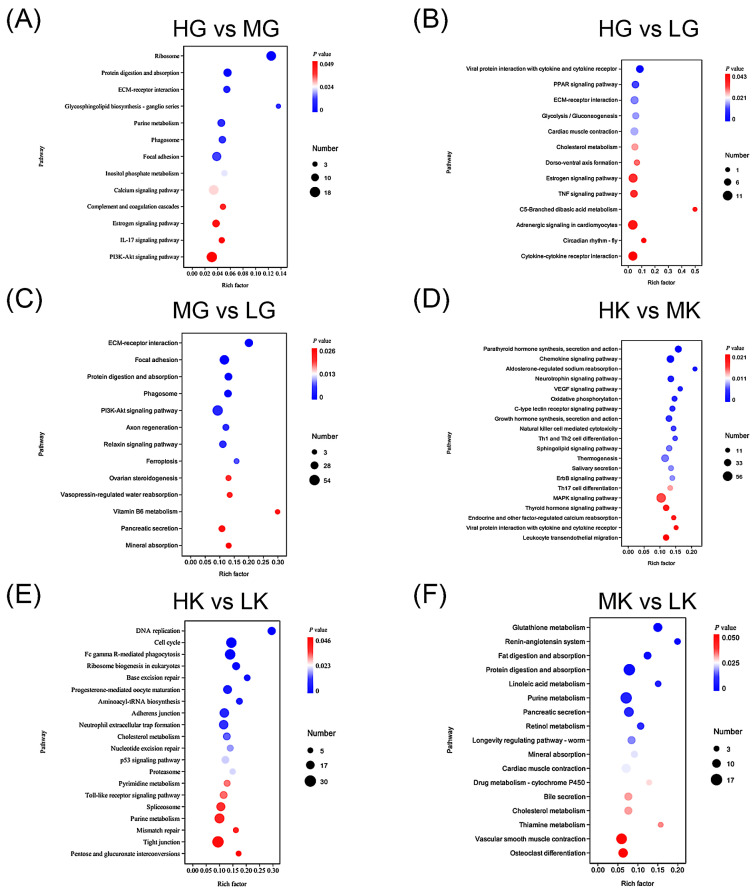
Enrichment analysis of DEGs KEGG in gill and kidney tissues under different pH conditions. Groups: gills (HG: pH 8.1, control; MG: pH 7.8; LG: pH 7.4) and kidneys (HK: pH 8.1, control; MK: pH 7.8; LK: pH 7.4). (**A**) Enrichment analysis of DEGs between HG and MG groups. (**B**) Enrichment analysis of DEGs between HG and LG groups. (**C**) Enrichment analysis of DEGs between MG and LG groups. (**D**) Enrichment analysis of DEGs between HK and MK groups. (**E**) Enrichment analysis of DEGs between HK and LK groups. (**F**) Enrichment analysis of DEGs between MK and LK groups. The *x*-axis represents the rich factor, and the *y*-axis represents the KEGG pathway. The size of the points indicates the number of differentially expressed genes enriched in the corresponding pathway, and the color intensity represents the level of significance.

The KEGG enrichment analysis results (Figure 5) highlight the pathways in which DEGs are significantly enriched across various groups. Specifically, the HG group compared to the MG group shows significant enrichment in protein-related pathways, such as the ribosome and protein digestion and absorption; metabolism-related pathways, including purine metabolism and glycerophospholipid biosynthesis; and immune-related pathways, like the IL-17 signaling pathway and phagosome (Figure 5A). These pathways are primarily involved in energy supply and the maintenance of internal homeostasis. In contrast, between the HG group and LG groups, DEGs are significantly enriched in immune-related pathways, such as viral protein interaction with cytokine and cytokine receptor, and the TNF signaling pathway; and metabolism-related pathways, including the PPAR signaling pathway and glycolysis/gluconeogenesis (Figure 5B). These pathways are crucial for immune defense and energy regulation. The comparison between the MG and LG groups reveals significant enrichment in pathways associated with cell adhesion, such as ECM–receptor interaction and focal adhesion; immune-related pathways, including the phagosome; and water and ion metabolism pathways, such as vasopressin-regulated water reabsorption and mineral absorption (Figure 5C). The water and ion metabolism pathways are particularly involved in ion exchange between the organism and its external environment, and fluctuations in the pH of culture water may be closely linked to these pathways.

For the comparison between the HK and MK groups, DEGs are significantly enriched in endocrine hormone-related pathways, including parathyroid hormone and growth hormone synthesis, secretion, and action; thyroid hormone signaling pathway; immune-related pathways, including chemokine signaling pathway, natural killer cell-mediated cytotoxicity, Th1, Th2, and Th17 cell differentiation; ion regulation-related pathways, such as aldosterone-regulated sodium reabsorption and calcium reabsorption; and metabolism-related pathways, including oxidative phosphorylation (Figure 5D). Endocrine hormone pathways play an important role in systemic regulation, oxidative phosphorylation pathways provide energy and participate in immune protection, while aldosterone-mediated sodium and calcium reabsorption pathways are essential for ion exchange regulation between the organism and the external environment. In the comparison between the HK and LK groups, DEGs are primarily enriched in DNA-related pathways, including DNA replication, cell cycle, base excision repair, pyrimidine metabolism, and purine metabolism (Figure 5E), all of which are involved in cellular damage repair. Finally, the comparison between the MK and LK groups shows significant enrichment in metabolism-related pathways, such as glutathione metabolism, digestion and absorption of fat and protein, purine metabolism, and mineral absorption, as well as water and ion metabolism-related pathways, including the renin–angiotensin system (Figure 5F). These pathways play a key role in maintaining physiological homeostasis, while the renin–angiotensin system is crucial for ion and water exchange between the gills, kidneys, and the external environment.

### 3.6. Sequencing Data Validation by qRT-PCR

**Figure 6:**
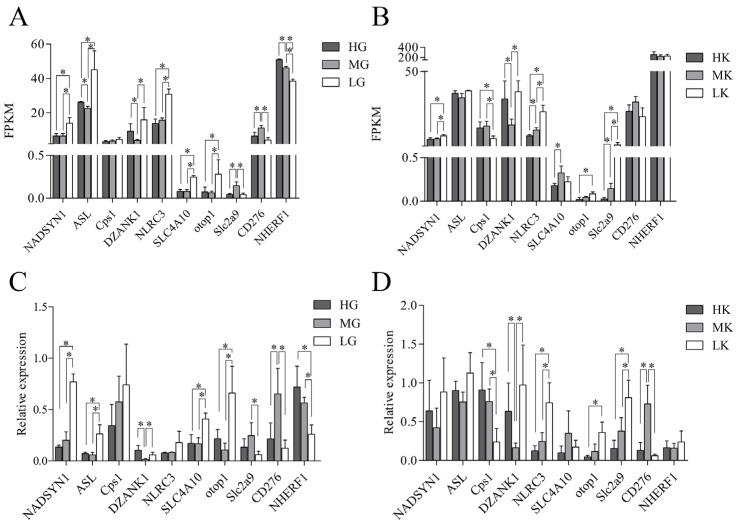
Gene expression changes revealed by RNA-Seq and qRT-PCR analyses. (**A**) Verification of gene expression levels in gills by RNA-Seq; (**B**) Verification of gene expression levels in kidneys by RNA-Seq; (**C**) qRT-PCR validation of gene expression in gills; (**D**) qRT-PCR validation of gene expression in kidneys. Groups: gills (HG: pH 8.1, control; MG: pH 7.8; LG: pH 7.4) and kidneys (HK: pH 8.1, control; MK: pH 7.8; LK: pH 7.4). The data of this study were presented as mean ± SD of three parallel measurements (*n* = 3). “*” above the lines indicate significant differences between groups (*p* < 0.05).

To verify the reliability and reproducibility of RNA-Seq data, we randomly selected 10 DEGs with significant expression differences between groups in the gill or kidney of *L. crocea* for qRT-PCR validation (Figure 6). The results showed that, in the gills, *NADSYN1*, *ASL*, *NLRC3*, *SLC4A10,* and *otop1* were significantly upregulated in the L group, while *CD276* and *NHERF1* were significantly downregulated. In the kidneys, *NLRC3* and *Slc2a9* were significantly upregulated in the L group, while *Cps* and *CD276* showed significant downregulation. The qRT-PCR results showed trends of upregulation and downregulation that were highly consistent with the RNA-Seq data, further confirming the reliability of the transcriptome sequencing data. Moreover, as the pH decreased, the expression level of *NHERF1* in the gills significantly decreased, whereas the expression of *NLRC3* and *Slc2a9* in the kidneys significantly increased.

## 4. Discussion

Due to severe acidification in coastal areas, these regions serve as natural laboratories to study the molecular responses of marine species as water pH declines, and to provide valuable insights into the mechanism of their acidification adaptation. This study focused on the large yellow croaker cultured in nearshore areas, and the results revealed that the physiological activities of *L. crocea* have a significant impact on the surrounding water’s pH levels. At pH 8.1, the water pH decreased; at pH 7.4, it significantly increased; while at pH 7.8, the water pH remained almost unchanged. These findings suggested that *L. crocea* may survive better under pH 7.8, indicating its long-term natural habitat. Furthermore, *L. crocea* were capable of regulating the water pH to a more suitable level for its survival. This phenomenon is not new, and our survey indicated that daily pH fluctuations in the farming areas of *L. crocea* were significantly higher than in the non-farming areas in the same region (data unpublished). Through controlled laboratory experiments, we further confirmed that the activities of *L. crocea* indeed altered the pH of the cultured water. In response to salinity stress, fish typically adopt conservative strategies, including excreting ions in high-salinity environments [48] and absorbing sodium and potassium ions in low-salinity conditions [49]. In response to acidification stress, it is traditionally considered that fish regulated the activity of acid–base balance-related enzymes to excrete acid and absorb alkali, thus maintaining internal acid–base homeostasis [16,50,51]. The excreting acid and absorbing alkali are both causing factors to decrease pH in water. However, we found that *L. crocea* adjusted water pH bidirectionally, indicating that a different adaptive strategy may be employed to cope with acidification stress.

Gills play a crucial role in various vital functions in fish, such as respiration, osmoregulation, and excretion [52]. As they maintain direct contact with the external environment, gills are particularly sensitive to environmental changes, especially concerning water quality. Moreover, gills are the primary organ involved in the acid–base regulation of fish [16,32,33]. Environmental stress can lead to significant alterations in the structure and the function of gills. Several studies have employed fish gills as indicators of environmental stress and harmful substances [53]. Consequently, histological and biochemical characters of the gills are commonly used as biomarkers to evaluate environmental suitability [54,55]. In the present study, we observed the shedding of epithelial cell in the gills under acidification stress, along with extensive apoptosis of chloride cells in the gill filaments. These changes indicated that acidification exerted significant stress on the gills of *L. crocea*. The further examination of oxidative-reduction-related enzyme activity in our study simultaneously supported this conclusion. In the L group, the MDA content was highest and significantly higher than that in the M group, suggesting that lipid peroxidation in the gills was increased under the pH 7.4. The acidic environment exacerbated gill stress, thus leading to the generation of a substantial amount of reactive oxygen species (ROS), which in turn promoted MDA production [56]. Moreover, SOD activity in the L group was significantly lower than in the M group, while no significant differences were observed in the activities of CAT and GSH-Px between the H and M groups. The deceased SOD levels may be attributed to the excessive ROS induced by stress. As a key antioxidant enzyme, the declined SOD activity impairs its ability to effectively scavenge free radicals, leading to oxidative damage of cell membranes and increased MDA production [57]. The reduced SOD activity revealed that the acidic environment under pH 7.4 exerted significant stress on the gills. Similarly, in the H group, the SOD activity was lower than that in the M group, but the MDA content was higher, which may be related to *L. crocea*’s needs to regulate the acid–base balance. At both pH 8.1 and 7.4, significant pH fluctuations in the culture water increased the metabolic burden on the gills. In contrast, the M group exhibited the lowest MDA content and the highest SOD activity, with nearly stable water pH, suggesting that the pH 7.8 had a minimal impact on the gill antioxidant enzyme system, resulting in milder physiological responses. This indicated that *L. crocea* was well-adapted to this pH condition. Notably, under pH 7.4, the lower water pH likely placed the gill in a state of overburdened metabolism, which may be the primary cause of the imbalance in the gill antioxidant defense system.

In addition, although the gills are the primary organ for the acid–base exchange in fish, the kidneys also play a crucial role in this process [36]. In this study, we observed mild turbidity, swelling, and degeneration in the renal tubules of the M group. In the L group, Bowman’s space disappeared, the kidney tissue structure loosened significantly, and the renal tubules showed noticeable signs of transparent degeneration. These changes are typical reactions of fish kidneys under environmental stress [58,59,60]. Enzyme activity assays indicated that the MDA content in the L group was significantly higher than that in the M and H groups, while the activities of SOD, CAT, and GSH-Px were notably lower in the L group compared to both the M and H groups. Similar studies have shown that, with a decrease in pH, the MDA content in the liver of yellowfin tuna significantly increased, while the SOD activity markedly decreased [61]. These observations indicated that acidic environments had a pronounced negative impact on the antioxidant defense system of fish and exacerbate lipid peroxidation. Furthermore, in this study, the antioxidant enzyme activities in the kidneys of both the H and M groups exhibited good stability, with the M group showing higher activity than the H group. This phenomenon may be attributed to the additional metabolic load imposed by the kidney’s involvement in regulating the pH of the surrounding water at pH 8.1. Overall, these results suggested that, under pH 7.4, the antioxidant enzyme activity and tissue structure of the kidneys in *L. crocea* were significantly negatively impacted, while the impact under pH 7.8 was relatively smaller.

This study utilized transcriptome sequencing technology to analyze the transcriptomic profiles of the gills and kidneys of *L. crocea* under different acidification conditions. The results revealed distinct molecular response patterns between the gills and kidneys. Significant differences in gene expression patterns in the gills were observed only under pH 7.4, while noticeable changes in the kidneys’ gene expression patterns occurred at pH 7.8. Furthermore, compared to pH 8.1, the number of DEGs in the gills was significantly lower than that in the kidneys. These findings revealed that the kidneys may be more sensitive to acidification than the gills in *L. crocea*. Previous studies have generally proposed that fish gills, being in direct contact with the surrounding water, rapidly accumulate H^+^ on the surface of the gill filaments when the water’s pH decreases. This accumulation interferes with the normal physiological functions of the gill cells [14,16,33]. In contrast, the kidneys primarily regulate the acid–base balance in the blood through the excretion and reabsorption of HCO_3_^3−^, a process that occurs more gradually [50]. Both acid excretion and alkali absorption are part of the passive defense mechanisms to maintain an internal acid–base balance in fish. Notably, in this study, the physiological activities of *L. crocea* resulted in significant changes in the pH of the culture water. This suggested that *L. crocea* may possess the ability to actively regulate water pH, potentially mediated by the kidneys. Fish kidneys can regulate the excretion of acidic or alkaline substances in the urine to maintain the internal acid–base balance [31,62]. Additionally, we observed that the number of DEGs between the MG and LG groups in the gills was significantly greater than that between the HG and MG groups (1543 vs. 481). In contrast, in the kidneys, the number of DEGs between the MK and LK groups was significantly fewer than that between the HK and MK groups (914 vs. 1841). This implied that the gills play a primary role in responding to low pH conditions, while the kidneys were more involved in managing high pH conditions.

We further conducted an enrichment analysis of DEGs in the gills and kidneys of *L. crocea* under different pH conditions. The KEGG annotation results showed that the DEGs in the HG and MG groups were mainly enriched in pathways related to physiological function maintenance and energy supply [63], such as ribosomes, protein digestion and absorption, and purine metabolism. This suggested that under pH 7.8, the gill cells of *L. crocea* regulated the expression of ribosome-related genes to adjust protein synthesis, digestion, and absorption, ensuring energy acquisition to adapt to the new pH environment. This is a common response of fish to environmental changes [64]. In addition, the DEGs between the HG and LG groups were mainly enriched in immune-related pathways, such as interactions between viral proteins and cytokines and receptors, TNF signaling pathways, as well as PPAR signaling pathways and glycolysis/gluconeogenesis related to energy supply [65]. This indicated that, compared to pH 8.1, pH 7.4 primarily affected immune function and energy supply in the gills of *L. crocea*. Studies have shown that aquatic animals typically reduce immune function and allocate more energy to adapt to environmental changes when responding to alterations in water conditions [66,67,68,69]. In this study, we also found that *NLRC3* was significantly upregulated, while *CD276* was significantly downregulated in the LG group. *NLRC3* is a negative regulator of innate immune responses [70], while *CD276* is an activator of T cell-mediated immune responses [71]. These findings implied that both innate and adaptive immunity were suppressed in *L. crocea* under pH 7.4. Furthermore, between the MG and LG groups, DEGs were significantly enriched in pathways related to substance exchanges [72], such as vasopressin-regulated water reabsorption and mineral absorption. Vasopressin not only stimulates and regulates Cl^3−^ secretion and H^+^ transport, but also regulates water reabsorption, playing an important role in ion and acid–base balance regulation in fish [72,73]. This finding indicated that *L. crocea* gills adapt well at pH 7.8, but when the pH drops to 7.4, the molecular mechanisms related to acid–base balance regulation were strongly activated. We also found that compared to the MG group, *ASL*, *SLC4A10*, and *otop1* were significantly upregulated in the LG group, while *slc2a9* and *NHERF1* were significantly downregulated. Fish are ammonium-excreting animals [74], and *ASL* is one of the key enzymes in the urea cycle, mediating nitrogen metabolism and preventing ammonia accumulation [75]. *SLC4A10* is a Na^+^/HCO_3_^3−^ cotransporter, mediating the influx and efflux of HCO_3_^3−^ and regulating cell pH [76]. *otop1* is a H^+^-selective ion channel that is bidirectionally regulated by acids and bases, mediating H^+^ influx and efflux under extracellular acid and base stimulation [77,78]. *SLC2A9* is a high-capacity urate transporter, which may play a role in the reabsorption of urate in the proximal renal tubules [79]. The Na^+^/H^+^ exchanger (NHE) is the primary Na^+^/H^+^ exchanger in fish, responsible for mediating the excretion of NH_4_^+^ [14]. These findings further suggested that under pH 7.4 conditions, the gills of *L. crocea* maintained internal environmental stability through an efficient and complex acid–base ion regulation mechanism. However, in contrast to previous studies [14,16], the NHE system in the gills was suppressed under acid stress, indicating that the process of H^+^ efflux from the gills was hindered. Under acidic stress, the increased cellular energy demand leads to the significant enrichment in metabolic pathways such as oxidative phosphorylation, resulting in elevated CO_2_ production [50]. This may account for the marked upregulation of *SLC4A10* and *otop1* expression in the gills, which are responsible for exporting CO_2_ in the form of HCO_3_^−^. This could be one of the potential reasons for the increase in pH in the cultured water.

In the kidneys, we found that DEGs between the HK and MK groups were primarily enriched in pathways related to hormone regulation and immune response, as well as in pathways such as aldosterone-regulated sodium reabsorption and endocrine, and other factor-regulated calcium reabsorption. Aldosterone plays a crucial role not only in regulating Na^+^ and K^+^ balance, but also in maintaining the acid–base balance in the body. When acid accumulates, aldosterone works synergistically with angiotensin II and other factors to stimulate renal acid excretion [80]. Furthermore, the expression of *slc2a9* in the HK group was significantly lower than that in the MK group, suggesting a reduced reabsorption of uric acid. These mechanisms may be critical for the adaptation of *L. crocea* to high pH conditions. Additionally, between the HK and LK groups, DEGs were primarily enriched in pathways related to cell proliferation, DNA replication, and damage repair, which was consistent with histological observations and the results of redox-related enzyme activities. This suggested that renal damage was the most severe under pH 7.4 conditions. In contrast, DEGs between the MK and LK groups were not only enriched in antioxidant-related pathways (such as glutathione metabolism and retinol metabolism) [81], but also in energy-related pathways involved in fat/protein digestion and absorption and purine metabolism. Moreover, they were significantly enriched in pathways related to fluid balance and electrolyte homeostasis, including the renin–angiotensin system (RAS) and mineral absorption. Similar results were observed in zebrafish, where exposure to acidified water significantly increased angiotensin II levels [82]. The RAS system plays a dominant role in sodium and water reabsorption in the kidney [83], and is activated in fish to respond to environmental stress, maintaining homeostasis through regulation of blood pressure and fluid balance [82,84,85]. Furthermore, *Cps* expression in the LK group was significantly lower than in the MK group, while *slc2a9* expression in the LK group was significantly higher than in the MK group. *Cps* participates in the urea cycle in the kidneys, catalyzing the conversion of bicarbonate and NH_4_^+^ into phosphate and H^+^ [86]. This indicated that under pH 7.4, the kidney may reduce acid excretion by inhibiting the production of H^+^ and enhancing uric acid reabsorption. These findings point to the fact that the kidneys also play a role in the adaptation of *L. crocea* to low pH environments.

Furthermore, we observed that as the pH decreased, the expression of *NHERF1* in the gills progressively declined, while the expression of *NLRC3* and *Slc2a9* in the kidneys increased. As the primary organ responsible for regulating the acid–base balance in the body, the gills account for over 90% of the regulation of extracellular pH. When the external environment becomes more acidic, the H⁺ excretion process in the gills is inhibited [14], with the highest H⁺ excretion activity occurring at pH 8.1 and the lowest at pH 7.4. This trend aligns with fluctuations in the pH of the aquaculture water. Based on these observations, we hypothesized that *NHERF1* plays a crucial role in the acid-base adaptation of *L. crocea*. Simultaneously, as environmental acidification increases, renal innate immune function is progressively suppressed, while the capacity for urate metabolism continues to be enhanced [70,79]. This phenomenon may be linked to increased purine metabolism, with the kidneys promoting the clearance of excess uric acid by reducing inflammatory responses and enhancing urate metabolism, thus helping to maintain normal renal function.

## 5. Conclusions

In conclusion, the results of the present study demonstrated that under pH 7.8 conditions, the pH of the aquaculture water remains stable, with the lowest MDA content observed in the gills and kidneys of *L. crocea*. Additionally, the activities of the antioxidant enzyme system (CAT, SOD, and GSH-Px) were higher compared to the pH 8.1 and pH 7.4 conditions. These findings suggested that the antioxidant enzyme system plays a crucial role in the adaptation of *L. crocea* to pH 7.8, helping to maintain homeostasis. Transcriptome analysis further indicated that the gills were primarily responsible for adapting to low pH environments, while the kidneys were mainly involved in adapting to high pH conditions. Specifically, metabolic pathways such as vasopressin-regulated water reabsorption, mineral absorption, aldosterone-regulated sodium reabsorption, and endocrine and other factor-regulated calcium reabsorption not only play a vital role in maintaining the acid–base balance in *L. crocea*, but may also mediate the processes of alkali excretion in the gills and acid excretion in the kidneys. qRT-PCR analysis of DEGs confirmed the accuracy of the RNA-Seq results. This study provided valuable insights into the large yellow croaker’s ability to adapt to different acidified water environments and offers a theoretical foundation for the management of coastal aquaculture, and for addressing future ocean acidification scenarios.

## Figures and Tables

**Table 1:** Statistical results of transcriptome sequencing of gill and kidney tissues of *L. crocea* under different acid challenges.

Tissues	Sample Name	Raw Reads	Clean Reads	Clean Data (bp)	Q20 (%)	Q30 (%)	GC (%)
Gill	HG1	49,790,102	48,817,232	7.35 G	98.40	95.77	46.05
	HG2	47,330,680	46,507,010	7.00 G	98.37	95.63	44.52
	HG3	59,354,782	58,282,594	8.78 G	98.49	95.98	46.25
	MG1	47,168,348	46,425,694	6.99 G	98.45	95.76	46.91
	MG2	56,646,552	55,621,006	8.38 G	98.49	95.95	46.50
	MG3	45,424,836	44,557,304	6.71 G	98.45	95.87	46.13
	LG1	62,603,754	61,397,376	9.23 G	98.37	95.78	43.76
	LG2	50,992,390	50,018,490	7.53 G	98.39	95.76	44.85
	LG3	36,776,506	36,168,694	5.44 G	98.60	96.29	45.96
Kidney	HK1	44,555,002	43,673,624	6.57 G	98.39	95.75	46.13
	HK2	48,738,750	47,954,210	7.22 G	98.64	96.39	46.04
	HK3	37,155,868	36,526,184	5.50 G	98.57	96.17	46.39
	MK1	49,127,630	48,366,024	7.29 G	98.45	95.69	47.35
	MK2	66,187,132	65,083,048	9.80 G	98.56	96.07	47.36
	MK3	54,160,660	53,111,080	8.00 G	98.42	95.77	48.01
	LK1	46,559,792	45,742,524	6.89 G	98.52	96.06	46.38
	LK2	56,608,916	55,531,990	8.36 G	98.47	95.89	46.90
	LK3	45,702,312	44,837,392	6.75 G	98.45	95.90	45.87

## Data Availability

The raw data of the present study have been submitted to NCBI with the following accession number: PRJNA1168280. All other data are contained within the main manuscript.

## Supplementary Materials

The following supporting information can be downloaded at: https://www.mdpi.com/article/10.3390/antiox14070872/s1, Table S1: Primers used in this study.
