# Supplementary material for: Adaptive Responses of Large Yellow Croaker Larimichthys crocea to Ocean Acidification: Integrative Analysis of Gill and Kidney Transcriptomics and Antioxidant Enzyme Activities
Source: Antioxidants (Basel). 2025 Jul 16;14(7):872. doi: 10.3390/antiox14070872 (PMC12291933; doi:10.3390/antiox14070872)
Supplement: Supplementary file 1 [file antioxidants-14-00872-s001.zip › antioxidants-3714944-supplementary.pdf]

Supplementary Table S1

Primers used in this study.

Gene	Nucleotide sequence (5’-3’)	
DZANK1	Forward	ACTGTCGGACTCTATTACCCA
	Reverse	TGCTTCCTCCAGTAACCTCT
NLRC3	Forward	CTATCCTGGACCGAAGC
	Reverse	TGCTGGTGAGGGTAGTT
CD276	Forward	GCTGACATCACTCCGTAT
	Reverse	CAACTGAGGCAGGGTC
NADSYN1	Forward	TAAGAGTCGTGGAGCCG
	Reverse	CTGGGTGATGGGAGATT
ASL	Forward	ATGCTGGCACAGACGG
	Reverse	TCCCACGAAGCGACCT
NHERF1	Forward	GGCTTCAATCTACGGG
	Reverse	CTTCCTGGCAACCTCA
Cps1	Forward	CTTGGCATTGTAGGCG
	Reverse	GCAGGCTGTGGTCTTCT
SLC4A10	Forward	CTGTGGTGGCGTTTG
	Reverse	TGCGGTCTTTAGCTTTAT
otop1	Forward	CAATGCCAGATAACACGC
	Reverse	TGTCTCCACGGCTTCC
slc2a9	Forward	ATGGATGCCCTACCTCA
	Reverse	CAGACCACCACAAACACC
β-actin	Forward	CCTTCACCACCACAGCCGAG
	Reverse	ATTCCGCAAGATTCCATACCGA
